# The differential response to neuronal hyperexcitation and neuroinflammation of the hippocampal neurogenic niche

**DOI:** 10.3389/fnins.2023.1186256

**Published:** 2023-07-11

**Authors:** Lorena Ruiz-Clavijo, Soraya Martín-Suárez

**Affiliations:** Achucarro Basque Center for Neuroscience, Leioa, Spain

**Keywords:** inflammation, neurogenic niche, neural stem cells, neuronal hyperexcitation, hippocampus

## Abstract

Hippocampal neurogenesis is a tightly regulated process in which neural stem cells (NSCs) get activated, enter in the cell cycle and give rise to neurons after a multistep process. Quiescent and activated NSCs, neural precursors, immature and mature neurons and newborn astrocytes coexist in the neurogenic niche in a strictly controlled environment which maintains the correct functioning of neurogenesis. NSCs are the first step in the neurogenic process and are a finite and, mostly, non-renewable resource, therefore any alteration of the intrinsic properties of NSCs will impact the total neurogenic output. Neuronal hyperexcitation is a strong activator of NSCs prompting them to divide and therefore increasing neurogenesis. However, neuronal hyperactivity is not an isolated process but often also involves excitotoxicity which is subsequently accompanied by neuroinflammation. Neuroinflammation normally reduces the activation of NSCs. It is technically difficult to isolate the effect of neuronal hyperexcitation alone, but neuroinflammation without neuronal hyperexcitation can be studied in a variety of models. In order to shed light on how the balance of neuronal hyperexcitation and neuroinflammation affect NSCs we analyzed proliferation and morphology of NSCs. We used two models of neuronal hyperactivity [an epilepsy model induced by KA, and a model of traumatic brain injury (TBI)] and different models of inflammation (LPS, Poly I:C, IFN-α and IL-6). We observed that only those models that induce neuronal hyperactivity induce NSCs activation but neuroinflammation causes the opposite effect. We also analyzed the response of other cell types in the neurogenic niche, focusing on astrocytes.

## Introduction

The hippocampus of most mammals, including the human hippocampus (Eriksson et al., 1998; Moreno-Jiménez et al., 2019), is able to keep generating new neurons postnatally, through adulthood and during aging through a complex process called adult hippocampal neurogenesis (AHN) (Altman and Das, 1965). AHN is a tightly regulated process in which neural stem cells (NSCs) located in the dentate gyrus get activated, enter into the cell cycle and give rise to neurons after a multistep process of neuronal progenitor division, survival and differentiation. In parallel, NSC-derived astrogliogenesis also takes place locally (Bonaguidi et al., 2011; Encinas et al., 2011). NSCs, neuronal precursors, immature and mature neurons and newborn astrocytes coexist in the hippocampal neurogenic niche in a tightly controlled environment which maintains the correct functioning of AHN.

NSCs are a finite and mostly non-renewable resource, and as a consequence, any alteration of the properties of NSCs will impact the total neurogenic output. Neuronal activity is a major regulator of neurogenesis which responds differentially to different levels of neuronal activity (Sierra et al., 2015; Bielefeld et al., 2019). In addition, while tonic gamma-aminobutyric acid (GABA) promotes the quiescence of NSCs, its reduction promotes their activation (entry into the cell cycle) (Song et al., 2012). Neuronal hyperexcitation (NHE) is a strong activator of NSCs (Hüttmann et al., 2003) and in extreme cases can trigger the generation of reactive-NSCs (React-NSCs) that abandon neurogenesis to generate reactive astrocytes (Sierra et al., 2015; Martín-Suárez et al., 2019). In contrast, neuroinflammation (NI) is a strong inhibitor of neurogenesis. A key common event process in-pathology and injury to the brain is neuroinflammation (NI), whose implication in the development of neurological and neurodegenerative disorders has become more prominent over the years. Although it is technically difficult to isolate the effect of NHE alone, NI without NHE can be studied in a variety of models. However, it must be kept in mind that NHE is not an isolated process as it frequently involves excitotoxicity (neuronal damage and cell death) which is subsequently accompanied by NI. We hypothesized that NI alone is not enough to trigger the induction of React-NSCs. To shed light on how the balance of NHE and NI affects NSCs we analyzed the rate of proliferation and the morphology of NSCs. We used three models of NHE: a model of epilepsy induced by a single intrahippocampal injection of a higher dose of kainic acid (HKA); a model of sub-seizure epileptiform activity induced by a single intrahippocampal injection of a lower dose of kainic acid (LKA) and a model of traumatic brain injury (TBI) induced by controlled cortical impact. In addition, we used three models of NI (LPS, Poly I:C, IFN-α or IL-6) (Nayak et al., 2014). We observed that only those models that induced NHE provoked NSCs activation and morphological changes. Our results confirmed that none of the NI stimuli induced a reactive phenotype in NSCs and that NSCs proliferation did not increase, or even decreased, due to NI, in agreement with previous studies. Besides the NSCs population, we also analyzed the response of astrocytes, as they are closely related to NSCs and the level of cell death induced in the neurogenic niche by NI and NHE models.

## Results

### NHE but not NI increases NSC division

**Figure 1:**
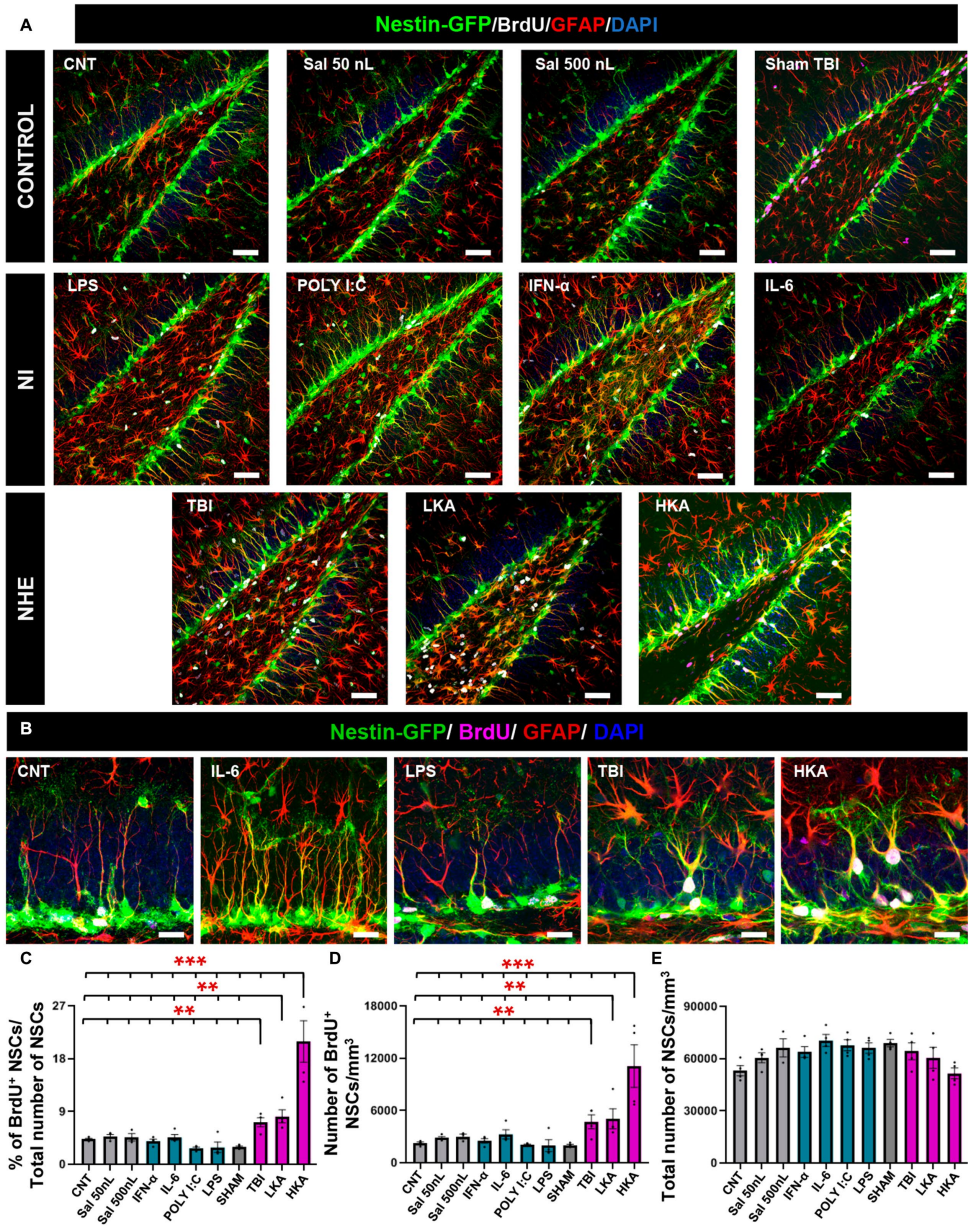
NHE but not NI induces NSCs hyperproliferation. **(A)** Confocal microscopy images showing the dentate gyrus of control, NI and NHE Nestin-GFP mice at 3 days after the procedure. Brain slices were immunostained for GFP, GFAP and BrdU. DAPI was used to stain cell nuclei. **(B)** Higher magnification images corresponding to representative models of NI and NHE. **(C)** Proportion of dividing NSCs among the total population of NSCs. **(D)** Density of BrdU^+^NSCs. **(E)** Total number of NSCs. The scale bar is 50 μm in **(A)** and 15 μm in **(B)**. *p < 0.05, **p < 0.01, ***p < 0.001. One-way ANOVA after all pairwise multiple comparisons by Holm-Sidak post hoc test. Bars show mean ± SEM. Dots show individual data.

To separate the effects of NHE from those of NI alone, we prepared a battery of models based on the administration of different agents to adult (3 months-old, m.o.) Nestin-GFP mice, in which NSCs can be readily visualized and analyzed (Mignone et al., 2004). In the NI models groups, in order to mimic viral and bacterial infections we administered the following agents: POLY I:C (polyinosinic: polycytidylic acid), a synthetic analog of double-stranded RNA (dsRNA) which is a molecular pattern associated with viral infections that stimulates the Toll-like receptor 3 (TLR3) pathway; LPS (lipopolysaccharides), a component of the outer membrane of bacteria that also stimulates TLRs; and the cytokines IL-6 (Interleukin-6) and IFN-α (interferon-alpha). As models of NHE, we used the controlled cortical impact version of TBI (Sullivan et al., 1998) and two doses of KA delivered by a single intrahippocampal injection as a model of epileptiform activity (LKA) and of mesial temporal lobe epilepsy (MTLE; HKA) (Sierra et al., 2015). The mice were subjected to an intrahippocampal injection of vehicle (saline, in different volumes) or POLY I:C, LPS, IL-6, IFN-α or LKA, HKA or to TBI on day 0. Control mice without any surgical manipulation were also added to compare with the potential local damage triggered by the cannula and the injection of the two different amounts of liquid (50 and 500 nL) used as a vehicle into the hippocampus (Figure 1A). On day 1 and on day 2 mice received 2 injections (6-h apart) of BrdU (150 mg/kg each). Mice were perfused on day 3 for analysis. Brain slices were immunostained for GFP, GFAP, and BrdU. DAPI was used as counterstaining (Figures 1A,B). To confirm the NHE effect of TBI we stained for C-Fos (Supplementary Figure S2A) and quantified the number of C-Fos^+^ cells in the GCL (Supplementary Figure S2B). To confirm the NI effect of POLY I:C, LPS, IL-6 and IFN-α we also immunostained for Iba-1 and BrdU (Supplementary Figure S3A) and quantified the number of Iba-1^+^ BrdU^+^ cells (Supplementary Figure S3B). Further, we confirmed the overexpression of cluster differentiation 68 (CD68), interleukin-1β (IL-1β) and tumor necrosis factor- (TNF-α) by quantitative PCR in dentate gyrus homogenates (Supplementary Figures S3C–E). For statistical differences see Supplementary Figure S4.

For the NHE groups (TBI, LKA, and HKA), each provoked a significant increment in NSCs mitotic activity compared to the controls and the NI groups. The mitotic activity was measured as the proportion of BrdU^+^NSCs among the total of NSCs (Figures 1A–C) and also as the density of BrdU^+^NSCs (Figure 1D). On the contrary, the NI models (POLY I:C, LPS, IL-6, IFN-α) caused a slight decrease or no effect on the mitotic activity of NSCs. The antiproliferative effect of the NI models was revealed when those groups were compared alone vs. the controls (Supplementary Figure S3F) as this effect was diluted by the dramatic action of the NHE models specially LKA. The overall number of NSCs was not altered by any treatment, as expected considering the short time between the application of the agent and the analysis (Figure 1E). Together, these results suggest that NSCs are more sensitive to NHE than to NI.

### NHE but not NI induces morphological changes in NSCs

**Figure 2:**
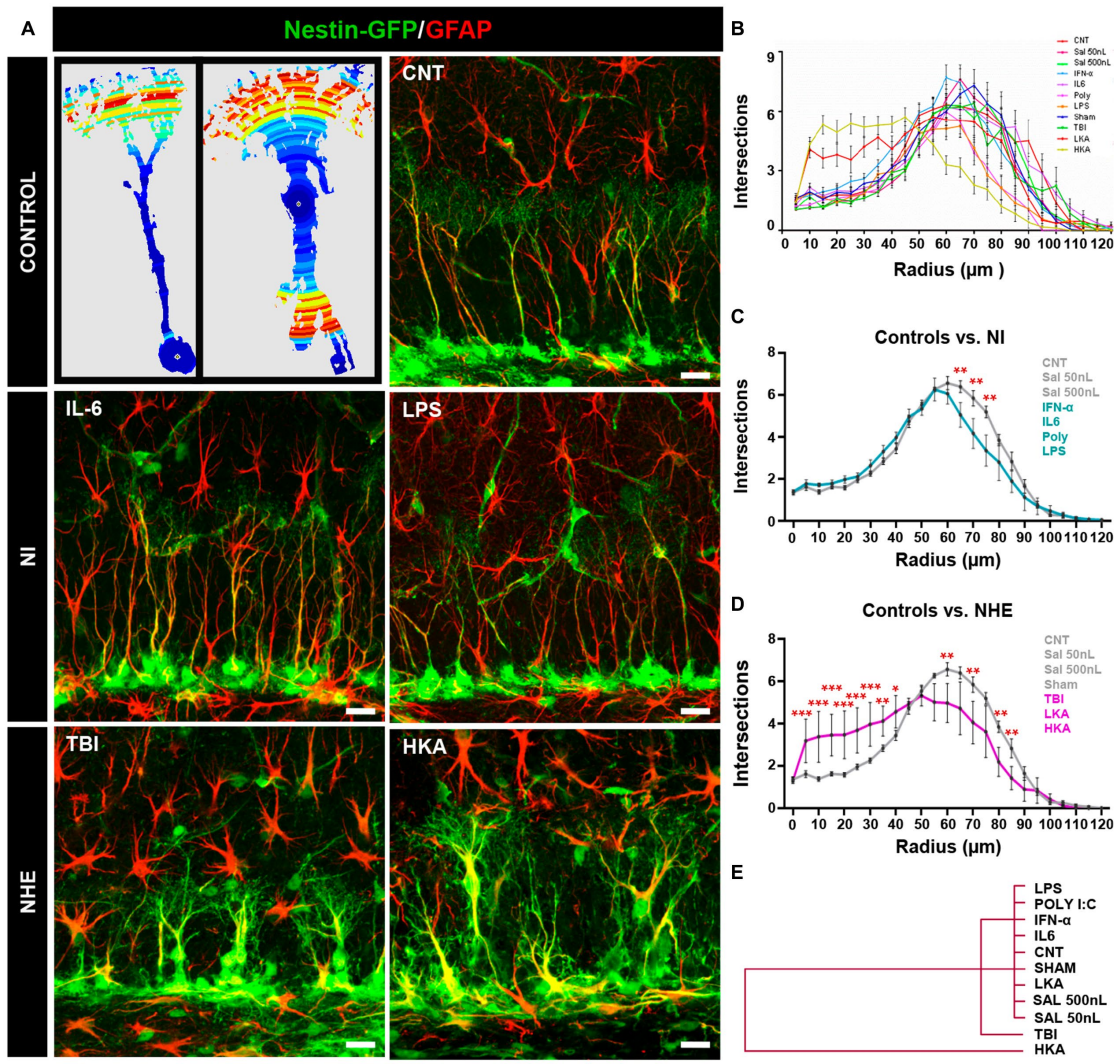
NHE but not NI induces morphological changes in NSCs. **(A)** The left panel shows examples of images originating from confocal microscopy-based z-stack projections of GFAP^+^Nestin-GFP^+^ NSCs and rendered after 3D-Sholl analysis. The color indicates the number of intersections in each radius (blue fewer and red, higher). The rest of the panels show confocal microscopy images showing NSCs in the SGZ at 3 days after the procedures. **(B)** Quantification of the morphological complexity of the NSCs based on the 3D-sholl analysis (number of intersections between the cell and virtual spheres of increasing radius). **(C)** Quantification of the morphological complexity of the NSCs pooling the NI models vs. controls. **(D)** Quantification of the morphological complexity of the NSCs pooling together the NHE models vs. the controls. **(E)** Plotting of Ward’s hierarchical clustering based on the complexity index (*K*-index) analysis to independently classify NSCs in each condition. **(B)** The scale bar is 10 μm in **(A)**. *p < 0.05, **p < 0.01, ***p < 0.00. ANOVA repeated measures followed by Bonferroni *post hoc* test in **(B–D)**.

Previous work has demonstrated that under high levels of neuronal hyperexcitation, such as epileptic seizures, over-proliferation of NSCs is linked to morphological changes and the transformation into React-NSCs, characterized by enlarged soma (hypertrophy) and the loss of the thin radial single process in favor of thicker multibranched prolongations (Sierra et al., 2015; Muro-García et al., 2019; Valcárcel-Martín et al., 2020). Interestingly this transformation does not take place when lower levels of NHE are induced, i.e., in non-convulsive epileptiform activity as mimicked by LKA (Sierra et al., 2015). We addressed whether the induction of the reactive phenotype of NSCs takes place only after seizures (mimicked by HKA) or it occurs also in TBI and NI models. We analyzed morphological changes in NSCs by 3D-sholl analysis using confocal microscopy-based 3D-reconstructions of NSCs (Figure 2A, upper left panel).

The overall comparison of all groups showed that whereas NI or LKA barely altered the morphological complexity of NSCs, TBI, together with HKA did (Figure 2B). The results of the statistical analysis are shown in Supplementary Figure S1A. For better visualization, we pooled and compared the NI groups vs. the control ones (Figure 2C) finding significant differences only in three of the twelve points of analysis (reflecting distance from the soma). In contrast, when we next pooled and compared the NHE groups vs. the control ones, NHE markedly presented more morphological complexity with statistical differences found in most of the points of analysis (Figure 2D). Of interest, TBI and HKA induced higher morphological complexity in the segments closer to the soma, reflecting the development of several processes emerging from the soma; and a reduction in the segments further from the soma, reflecting the loss of the profuse and fine broccoli-like arborization typical of normal NSCs.

We also analyzed the Sholl-regression coefficient (k-index) which measured the rate of decay of complexity (number of intersections) in relation to the distance to the soma (Figure 2E). The analysis classified NSCs into three differentiated groups. NI models and controls clustered together, reflecting the fact that NI alone did not affect the morphology of NSCs. Closer to the control+NI cluster, but independent from it, are the NSCs corresponding to the TBI model. Finally, the cluster furthest from controls and NI was the one formed by NSCs corresponding to the HKA model. These results show that in a similar fashion to over-proliferation, only NHE, particularly TBI and HKA, induced a reactive-like morphological phenotype in NSCs.

### NHE and NI can increase astrogliosis in the neurogenic niche

**Figure 3:**
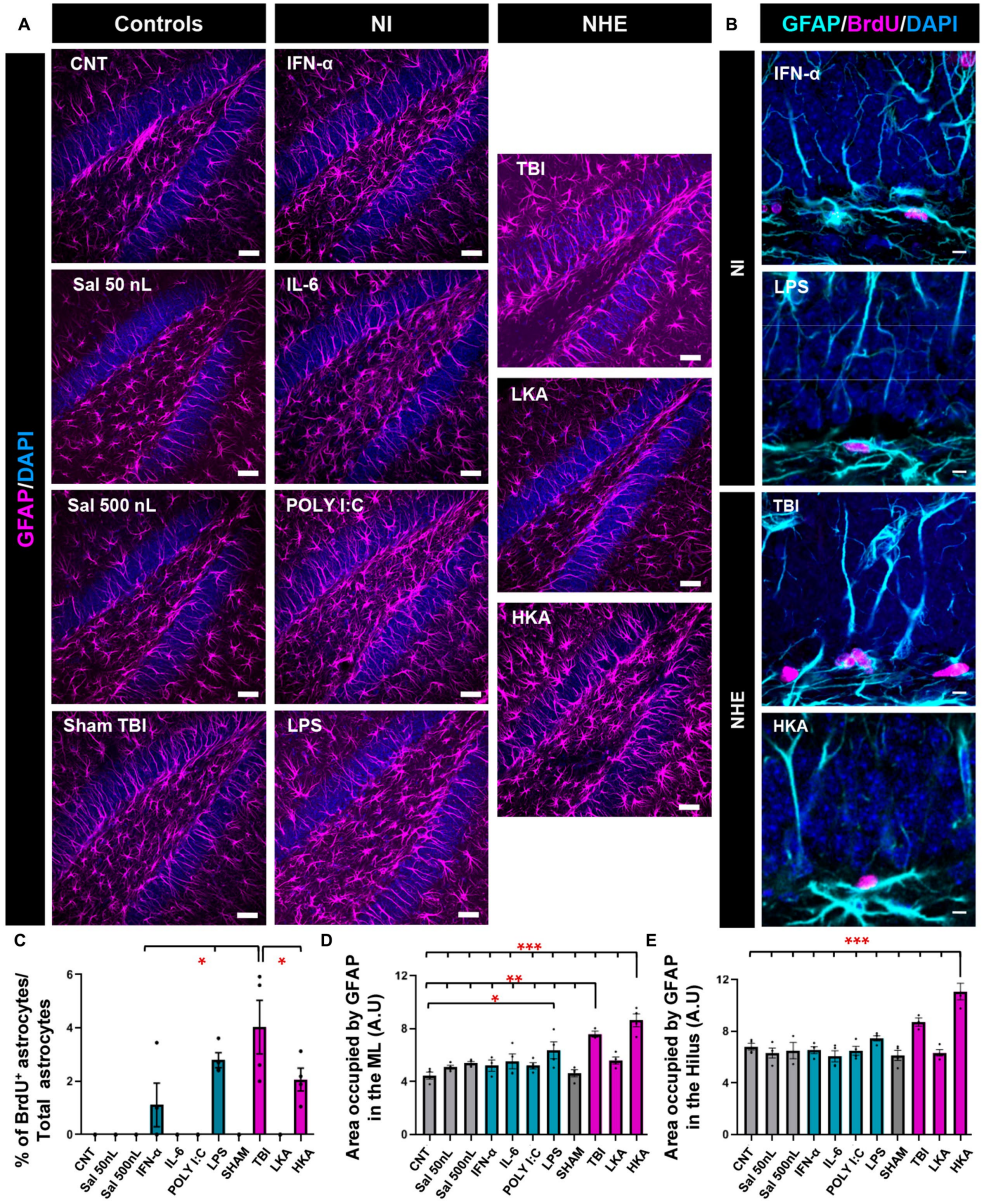
NHE and NI can increase astrogliosis in the neurogenic niche. **(A)** Confocal microscopy images showing immunostaining for GFAP in the dentate gyrus. **(B)** Higher-magnification confocal microscopy images of dividing GFAP^+^ (Nestin-GFP-negative) cells in the GCL+SGZ after immunostaining for GFAP and BrdU. DAPI was used to stain cell nuclei in **(A)** and **(B)**. **(C)** Proportion of dividing GFAP^+^ cells among the total population of GFAP^+^ cells. **(D)** Percentage of the area occupied by GFAP^+^ pixels in the molecular layer. Only GFAP^+^ cells that were Nestin-GFP-negative were counted. Nestin-GFP is omitted for better visualization. **(E)** Percentage of the area occupied by GFAP^+^ pixels in the hilus. The scale bar is 25 μm in **(A)** and 10 μm in **(B)**. *p < 0.05, **p < 0.01, *p < 0.001 one-way ANOVA after all pairwise multiple comparisons by Holm-Sidak post hoc test. Bars show mean ± SEM. Dots show individual data.

We next sought to test whether astrocytes responded in the same manner as NSCs (Figure 3) in the DG. We first checked astrocytic proliferation in the GCL+SGZ by quantifying the proportion of astrocytes (defined as Nestin-GFP-negative, GFAP^−^ positive cells) that incorporated BrdU (Figure 3A). The Nestin-GFP channel (see Figures 1A,B) has been omitted for better visualization. Overall, astrocytic proliferation was not altered except for two models of NI (IFN-α and LPS) and two more extreme models of NHE (TBI and HKA). In these cases, a significant increase in the percentage of BrdU^+^ astrocytes was found (Figures 3B,C). However, this increase is very minor in total numbers and stands out because in the rest of the conditions, astrocyte division was virtually non-existent. Despite this argument, the response of astroglia is different from that of NSCs, because NSCs do not increase their mitotic activity in response to any of the NI challenges (Figure 1). We next evaluated the development of astrogliosis in the dentate gyrus by quantifying the area occupied by GFAP^+^ pixels (Figure 3A) in the molecular layer and the hilus, avoiding the GCL and the SGZ so that NSCs were excluded from the analysis. Considering that astrocytes hardly divide, any increase in GFAP coverage can be attributed to hypertrophic cell morphology rather than to an increased number of astrocytes. The expression of GFAP was significantly increased in the ML only in the TBI and HKA models (Figure 3C). The same result was obtained for the analysis of the hilus (Figure 3D).

### NHE and NI can cause cell death

**Figure 4:**
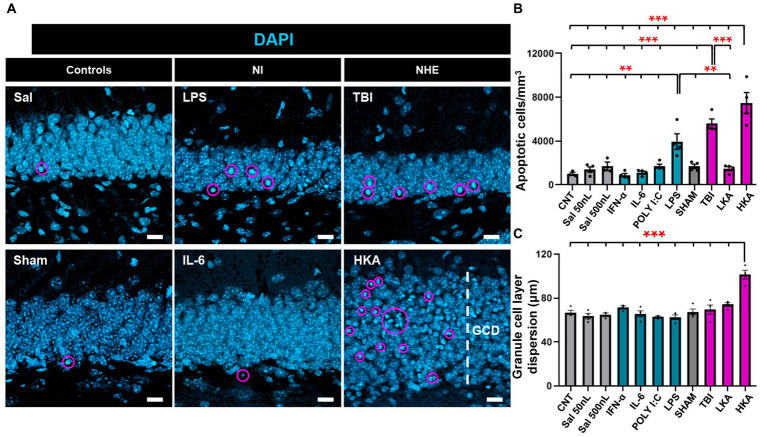
NHE and NI can cause cell death. **(A)** Confocal-microscopy images of the GCL stained for DAPI. DAPI DNA staining was used to identify apoptotic (pyknotic and karyorrhexic nuclei). **(B)** Quantification of the number of apoptotic nuclei. **(C)** Quantification of the GCL thickness, measured as the distance between the hilus and the molecular layer. **p < 0.01, ***p < 0.001 one-way ANOVA after all pairwise multiple comparisons by Holm-Sidak posthoc test Bars show mean ± SEM. Dots show individual data. The scale bar in **(A)** is 10 μm.

Finally, we checked the effect of the different models of NI and NHE on cell death in the neurogenic niche. First, we checked the number of apoptotic cells in the GCL+SGZ (Figure 4A). The number of apoptotic cells was assessed by DAPI staining and defined as the number of condensed nuclei (pyknotic/karyorrhectic nuclei) (Figure 4A). This manner of quantifying cell death is more reliable than those based on the expression of markers (Sierra et al., 2010), as in dying cells protein degradation varies greatly from earlier to later stages of the apoptotic process. Apoptosis was significantly increased in two of the NI models (LPS and POLY I:C) and two of the NHE models (TBI and HKA) (Figure 4B). As an additional readout of alterations induced in the dentate gyrus by pathological conditions, we measured the dispersion of the GCL (GCD) (Figure 4A), an important marker in epilepsy. In agreement with previous data, we only observed an increase in the thickness of the GCL in HKA (Figure 4C). These results imply that NHE induces a stronger response in the neurogenic niche than inflammation.

## Discussion

Adult hippocampal neurogenesis is a highly plastic process that fluctuates depending on the state of its environment. Local vasculature, neurons, microglia and astrocytes interact and modulate the process of neurogenesis (Palmer et al., 2000; Ge et al., 2006; Sierra et al., 2010; Sultan et al., 2015). It comes as no surprise that neurogenesis is directly affected in situations in which the neurogenic niche is disrupted by alterations in one or several of those cell types. NI, as found in models of infection or neurodegenerative disorders, typically inhibits cell proliferation and neurogenesis. On the other side, situations that involve NHE, such as epileptiform activity or TBI tend to consistently increase activation of NSCs and neurogenesis, at least in the short term (Sun, 2004; Sun et al., 2007; Das and Basu, 2008). We herein focused on hippocampal NSCs as they are the first step of the neurogenic cascade, acting as a mostly non-renewable source of new neurons (Bonaguidi et al., 2011; Encinas et al., 2011; Pilz et al., 2018). The wide range of their functional capabilities as well as their responses to changes in the environment is still not fully understood. The plasticity and variety of responses of NSCs are of special interest in pathological conditions in which they could be acting as biosensors of disease and damage (Rodríguez-Bodero and Encinas-Pérez, 2022), a hypothesis that we wanted to investigate further. We therefore herein compare side-to-side, and in an acute setting, the alterations produced on NSCs by different acute models of NI and NHE.

We have shown before that seizures, in an experimental model of MTLE, induce profound changes in NSCs, namely, increased activation (entry into mitosis) and the development of a reactive morphology, two readouts that can be readily used to test the response of NSCs to different stimuli. Interestingly both are graded and can be used quantitatively to measure the level of response of NSCs. For instance, the reactive-like phenotype, characterized by cell hypertrophy and development of a multibranched morphology, is maximal in an experimental model of MTLE with hippocampal sclerosis (Sierra et al., 2015; Muro-García et al., 2019; Valcárcel-Martín et al., 2020), but is less dramatic in a mouse model of Dravet Syndrome, a generalized epilepsy (Martín-Suárez et al., 2020). As we show here, this scenario is also the case for the controlled cortical impact used as a model of TBI. A low dose of KA mimicking interictal epileptiform activity (without seizures of any class) triggers NSC activation but does not alter cell morphology.

Conditions that involve high levels of NHE such as MTLE and TBI are accompanied by NI due to neuronal excitotoxicity. Our main objective in this work was to test whether these changes in NSCs could be attributed to NI alone. Previous literature shows that NI tends to inhibit NSCs activation (reviewed in Das and Basu, 2008), but the effect on morphological reactivity had not been addressed. As it is difficult to separate NI from NHE we employed a battery of NI-alone models to compare with NHE models. Of note, we focused on an acute paradigm because the effects triggered by NHE tend to occur in a rapid manner (24 h to 3 days), as shown after the injection of HKA, used as the main reference model (Sierra et al., 2015; Muro-García et al., 2019; Valcárcel-Martín et al., 2020).

NI can be triggered by a variety of stimuli including pathogens, such as bacteria, fungi, parasites, and viruses. To cover a wide range of models we resorted to mimic bacterial infection with LPS and viral infection with Poly I:C. In addition, we directly delivered cytokines (IFN-α or IL-6). These factors are frequently administered intraperitoneally which triggers a barrage of systemic actions that we wanted to avoid. We, therefore, resorted to injecting these factors directly into the hippocampus, allowing also a more direct comparison with the results obtained with the intrahippocampal injection of KA used as a standard model of MTLE.

It has been described that NSCs express mRNA that encodes the TLR family (Rolls et al., 2007) thus NSCs could react directly to LPS. On the contrary, Poly I:C, used to mimic a viral-infection-derived inflammation process, has not been reported to act directly on NSCs. We also injected into the hippocampus cytokines such as IL-6 or IFN-α, effective mediators of the inflammatory process itself. We observed that neither NSCs activation nor morphology was significantly altered. Nevertheless, the anticipated inactivating effect of the NI challenges was unveiled when testing the effect of NI vs. controls alone, without the dramatic pro-activation effect of NHE. These results are in concordance with previous studies showing that NSC activation, as well as cell proliferation and neurogenesis are reduced by interleukins, interferons and TNF-α (reviewed in Das and Basu, 2008). Our acute setting contrasts with previous models in which the effect is tested after longer periods and systemic administration is used (Kaneko et al., 2006; Zheng et al., 2014), which can result in an amplification of the neuroinflammatory cues getting to NSCs. Other previous studies had demonstrated the anti-neurogenic effect of different models of inflammation, although without dwelling in detail on the effect of NSCs (Sun, 2004; Widera et al., 2006; Nakanishi et al., 2007; Ben-Hur, 2008).

We used two models of NHE to test the effect on NSCs, the standardized model of intrahippocampal injection of KA, which offers the advantage of playing with different doses to mimic either seizures or epileptiform activity alone, and controlled cortical impact as a model of TBI. In TBI and HKA we found increased activation of NSCs as well as induction of the reactive phenotype. In the LKA model, only activation of NSCs was found to be augmented, showing that both responses are independent and that the reaction of NSCs to changes in the niche can be fine-tuned. Triggering an increase in NSCs activation would require a lower threshold of NHE than the morphological changes. The mechanisms that translate NHE into higher NSC activation or morphological changes remain to be elucidated, as well as the functional significance of the development of the reactive-like phenotype. Due to the close relationship between NSCs and astrocytes, we also tested the effect of the NI and NHE models in this cell type. Our results showed that also the astrocytic response is higher under NHE than in NI in our acute setting, although in some of the NI models there was an increase in astrocytic proliferation. IFN-α and LPS cause an increase in astrocyte cell division, however, in actual numbers, this effect is very small, as astrocytic proliferation in basal conditions is virtually non-existent. Still, this result, together with the overexpression of GFAP, highlights the different behavior of NSCs and astrocytes to changes in the environment and suggests that NSCs are more sensitive than astrocytes to changes in the niche. Our data agree with previous results showing how NSCs from the subventricular zone are very sensitive to fluctuations in the local and systemic milieu (Belenguer et al., 2021). Overall, our results suggest that NHE tends to trigger a stronger effect on NSCs than NI and that NHE can overpower NI when both processes take place at the same time.

## Material and methodology

### Animals

Nestin-GFP transgenic mice were used in all the experiments. In Nestin-GFP mice, GFP is expressed under the regulatory elements of the intermediate filament Nestin, expressed in neural stem and progenitor cells. All the animals were housed with *ad libitum* food and water access, in a 12:12 h light cycle. Nestin-GFP transgenic mice, kindly provided by Dr. Grigori Enikolopov at Cold Spring Harbor Laboratory (Cold Spring Harbor, NY, United States), were crossbred with C57BL/6 mice for at least 10 generations (Mignone et al., 2004). All procedures on these mice were approved by the University of the Basque Country (EHU/UPV) Ethics Committee (Leioa, Spain) and the Comunidad Foral de Bizkaia (CEEA: M20/2015/236 and M20/2022/129). All procedures followed the European directive 2010/63/UE and NIH guidelines. The mice were 3 months old. The number of animals used in each experiment was 4.

### BrdU administration

The labeling of proliferating cells, progressing through S-phase, was performed by intraperitoneal injection of BrdU. BrdU (5-Bromo-1-(2-deoxy-β-D-ribofuranosyl) uracil, 5-Bromouracil deoxyriboside; Sigma, St Louis, MO, United States, REF: B5002) was diluted in sterile saline at 150 mg/kg. Mice received 2 injections separated by 6 h on the first and the second day after the surgery and sacrificed on the third day post-surgery.

### NI models

Three months old mice were anesthetized with intraperitoneal ketamine (Ketolar, Pfizer)/xylazine (Sigma) (10:1 mg/kg) and received a single dose of the analgesic buprenorphine (1 mg/kg) (Buprecare, Animalcare Ltd) subcutaneously. The hair over the scalp was shaved and povidone-iodine was applied to clean and disinfect. After positioning in the stereotaxic apparatus, a 0.6 mm hole was drilled at coordinates taken from Bregma: anteroposterior (AP) −1.8 mm, laterolateral (LL) −1.6 mm. A pooled glass microcapillary was inserted at −1.9 mm dorsoventral (DV). Inflammation was induced by the administration of different drugs. To mimic bacterial infection LPS (8 mg/mL; Sigma) was injected. Poly I: C (2 μg/μl; Invivogen) was used to induce viral infection. To stimulate the immune response IFN-α (4*10^5^ IU/Kg; Miltenyi Biotec) was administered and IL-6 (IL-6 mouse recombinant 1 mg/mL; 10 μg) was used to increase the levels of cytokines in the dentate gyrus.

### NHE models

We followed the protocol optimized and described in Sierra et al. (2015), Abiega et al. (2016), Martín-Suárez et al. (2019), and Bielefeld et al. (2019). 50 nL of kainic acid (KA) (Sigma, St Louis, MO, United States, REF: K0250) was administered at 0.74 mM (Low Kainate-LKA) and 20 mM (High Kainate-HKA) (Bouilleret et al., 1999; Sierra et al., 2015). The single injection was done following the same procedure and using the same coordinates as described above for the NI agents.

For traumatic brain injury (TBI) (Sullivan et al., 1998) mice were positioned in the stereotaxic apparatus, and a midline incision was made in the skin over the scalp. Next, a 4 mm craniotomy was made lateral to the sagittal suture and centered between bregma and lambda. The skull cap was removed without damaging the dura. The pneumatically controlled contusion was driven by an impactor fitted with a rounded stainless-steel tip of 3 mm in diameter (Precision Systems and Instrumentation, Fairfax, VA). The impact compressed the cortex to a depth of 0.5 mm (mild) or 1.0 mm (severe) at a velocity of 3.5 m/s and 400 ms duration. After the surgery, the skull cap was replaced and the mice were sutured.

Control animals were injected with 50 nL or 500 nL of saline (Sal). An additional pure control was added to compare the possible effect of the injection. All the experiments were performed in the right hippocampus using a microinjector (Nanoject II, Drummond Scientific, Broomal, PA, United States). After 2 min, the microcapillary was retracted, and the mice were sutured and maintained in a thermal blanket until recovered from anesthesia. The animals were monitored during the hours following the procedure.

### Immunohistochemistry

Immunohistochemical techniques, image capture, and quantification techniques were performed essentially as described before following methods optimized for the use in transgenic mice (Encinas, 2008; Encinas et al., 2011; Sierra et al., 2015; Muro-García et al., 2019; Martín-Suárez et al., 2019, 2020). Animals were transcardially perfused with 20 mL of PBS followed by 20 mL of 4% (w/v) paraformaldehyde (PFA) in PBS. The brains were removed and postfixed for 3 h in the same fixative, then the brains were transferred to PBS and kept at 4°C. Serial 50 μm-thick sagittal sections were cut using a Leica VT 1200S vibratome (Leica Microsystems GmbH, Wetzlar, Germany).

Immunostaining was performed following a standard procedure: the sections were incubated with blocking and permeabilization solution [0.25% Triton-X100 and 3% bovine serum albumin (BSA)] in PBS for 3 h at room temperature, and then incubated overnight with the primary antibodies (diluted in the same blocking and permeabilization solution) at 4°C.

Before incubating with the secondaries, sections were washed with PBS. The sections were incubated with fluorochrome-conjugated secondary antibodies diluted in the same permeabilization and blocking solution for 3 h at room temperature.

The sections were mounted on slides with Dako fluorescent mounting medium (Agilent-Dako S3023).

The following primary antibodies were used: chicken anti-GFP (Aves laboratories, GFP-1020, 1:1000), rat anti-BrdU (Bio-Rad, MCA2060, 1:400), rabbit anti-GFAP (Dako, Z0334, 1:1000), rabbit anti-C-Fos (Santa Cruz sc-7202, 1:1000), rabbit anti-Iba 1 (Wako 19-19741, 1:1000).

The secondary antibodies used, all from ThermoFisher Scientific and in 1:500 concentration, were: goat anti-chicken Alexa Fluor 488 (ThermoFisher Scientific, A11039), donkey anti-rat Alexa Fluor 594 (ThermoFisher Scientific, A21209), donkey anti-rabbit Alexa Fluor 680 (ThermoFisher Scientific, A10043). 4′,6-diamidino-2-phenylindole (DAPI, Sigma-Aldrich), at 1:1000, was also added to the sections during the incubation with the secondary antibodies to counterstain cell nuclei.

### Image capture

All fluorescence immunostaining images were collected using a Leica SP8 laser scanning (LAS X software). The signal from each fluorochrome was collected sequentially, and controls with sections stained with single fluorochromes were performed to confirm the absence of signal leaking into different channels and antibody penetration. All the images were adjusted equally for the entire image using the “brightness and contrast” and “levels” controls from the “image/adjustment” set of options without any further modification. All images shown are projections from z-stacks ranging from 10 (typically for individual cell images) to 20 microns of thickness.

### Cell quantification

Quantitative analysis of cell populations (proliferation) in Nestin-GFP mice was performed by design-based (assumption-free, unbiased) stereology using a modified optical fractionator sampling scheme as previously described (Encinas, 2008; Encinas et al., 2011). Hemispheres were sliced sagittally. Slices were collected using systematic-random sampling. The 50-μm slices were collected in 5 parallel sets, each set consisting of approximately 14 slices, each slice 250 μm apart from the next. Cells were categorized following the criteria described previously (Encinas, 2008; Encinas et al., 2011; Sierra et al., 2015; Muro-García et al., 2019; Martín-Suárez et al., 2019, 2020).

NSCs were defined as radial glia-like Nestin-GFP^+^ GFAP^+^ cells with the cell body located in the subgranular zone (SGZ) or the lower third of the granule cell layer (GCL) and with an apical process extending from the SGZ toward the molecular layer (ML) through the GCL. Astrocytes were defined as GFAP^+^ Nestin-GFP^−^ cells.

The relative proportions of dividing NSCs were identified as Nestin-GFP^+^, GFAP^+^, BrdU^+^ NSCs among the total number of NSCs population. Dividing astrocytes were referred to as GFAP^+^, BrdU^+^ and Nestin-GFP^−^ cells among the total population. Granule cell dispersion was measured in DAPI-stained sections. The proportion of dividing microglia was identified as Iba1^+^BrdU^+^ cells among the total Iba+ population. The density of C-Fos cells was quantified as of the number of C-Fos positive cells in the GCL+SGZ per mm3. The thickness of the GCL was measured in at least 2 points in each slice. At least 5–6 slices were analyzed.

### Gliosis

The area occupied by astrocytes was measured in the ML and the hilus separately, using the open-source FIJI (Image J). First, GFAP^+^ cells (but Nestin-GFP-negative) were manually selected in z-stacks using the “threshold” tool to outline only the pixels of the image with GFAP^+^ staining. Using the “Measure” tool we calculated the area fraction measure as Those pixels that are highlighted using the threshold tool (in arbitrary units). Area fraction was calculated from at least 5 z-stack of 12 μm of thickness. A minimum of five hippocampal sections were analyzed per animal.

### 3D-Sholl analysis

3D-Sholl-analysis was performed as previously described (Martín-Suárez et al., 2019). At least 40 individual cells were analyzed. NSCs were captured with the 63x objective, by a confocal microscope, making sure they contained the whole cell. We used the 3D-Sholl open-source plug-in for FIJI (Image J) (Schindelin et al., 2012). This plug-in performs the 3D-Sholl technique directly on z-stacks of fluorescence-labeled cells (detailed in the user guide of http://fiji.sc/Sholl).

### Quantification of apoptosis

Apoptosis was quantified as previously described (Abiega et al., 2016; Martín-Suárez et al., 2020). Apoptosis was assessed by DAPI staining and quantification of the number of pyknotic/karyorrhectic (condensed/fragmented DNA) nuclei was carried out.

### RNA isolation from dentate gyrus

The injected (right) dentate gyrus of Nestin-GFP mice was rapidly isolated in cold HBSS. Using a roto-stator homogenizer and Qiagen RNeasy Mini Kit, RNA was isolated following the manufacturer’s instructions, including a DNAse treatment to eliminate genomic DNA residues. The quantity of RNA was measured using the Nanodrop 2000. Next, 1.5 μg of RNA were retro-transcribed using random hexamers (Invitrogen) and Superscript III Reverse Transcriptase kit (Invitrogen), accordingly to the manufacturer’s instructions in a Veriti Thermal Cycler (Applied Biosystems, Alcobendas, Spain).

### RT-qPCR

RT-qPCR was performed following MIQE guidelines (Minimal Information for Publication of Quantitative Real-Time Experiments) (Bustin et al., 2009). Three replicas of 1.5 μL of a 1:3 dilution of cDNA were amplified using Power SybrGreen (Biorad) for dentate gyrus experiments or three replicas of 1.5 μL of a 1:2 dilution of cDNA were amplified using SoFast EvaGreen Supermix (Biorad) for isolated NSCs experiments in a CFX96 Touch Real-Time PCR Detection System (Biorad). The amplification protocol for both enzymes was 3 min 95°C, and 45 cycles of 10 s at 95°C, and 30 s at 60°C.

### Primers

Primers were designed to amplify exon-exon junctions using Primer Express (Thermo Fisher Scientific) or using the Predesigned primers for gene expression analysis (KiCqStart^®^ SYBR^®^ Green Primers) from Sigma. Table 1 shows the sequences of the different Primers.

**Table 1:** PRIMERS.

Primer	Reference	Sequence
HPRT	NM_013556.2	Fwd ACAGGCCAGACTTTGTTGGA Rev. ACTTGCGCTCATCTTAGGCT
IL-1β	NM_008361	Fwd CAACCAACAAGTGATATTCTCCATG Rev. GATCCACACTCTCCAGCTGCA
CD68	NM_001291058.1	Fwd CAATTCAGGGTGGAAGAAAG Rev. TCTGATGTAGGTCCTGTTTG
TNF-α	NM_013693	Fwd CATCTTCTCAAAATTCGAGTGACAA Rev. TGGGAGTAGACAAGGTACAACCC

### Statistical analysis

SigmaPlot (San Jose, CA, United States) was used for statistical analysis. One Way ANOVA was performed in all cases to compare data. To analyze the data from the 3D-Sholl analysis we employed a repeated-measures two-way ANOVA with a Bonferroni *post-hoc* test. Only *p* < 0.05 is reported to be significant. Data are shown as mean ± standard error of the mean (SEM).

## Data availability statement

The raw data supporting the conclusions of this article will be made available by the authors, without undue reservation.

## Ethics statement

The animal study was reviewed and approved by all procedures on mice were approved by the University of the Basque Country (EHU/UPV) Ethics Committee (Leioa, Spain) and the Comunidad Foral de Bizkaia (CEEA: M20/2015/236 and M20/2022/129). All procedures followed the European directive 2010/63/UE and NIH guidelines.

## Author contributions

SM-S: conceptualization, formal analysis, data curation, manuscript writing, preparation, and visualization. SM-S and LR-C: methodology and validation. All authors contributed to the article and approved the submitted version.

## Funding

This work has been funded by the MINECO (with FEDER Funds) SAF2-015-70866-R, MICINN PID2019-104766RB-C21, and MICINN RyC2021-033215-I. LR-C is a Basque Government predoctoral contract awardee.

## Conflict of interest

The authors declare that the research was conducted in the absence of any commercial or financial relationships that could be construed as a potential conflict of interest.

## Publisher’s note

All claims expressed in this article are solely those of the authors and do not necessarily represent those of their affiliated organizations, or those of the publisher, the editors and the reviewers. Any product that may be evaluated in this article, or claim that may be made by its manufacturer, is not guaranteed or endorsed by the publisher.

## References

[ref1] Abiega O. Beccari S. Diaz-Aparicio I. Nadjar A. Layé S. Leyrolle Q.. (2016). Neuronal hyperactivity disturbs ATP microgradients, impairs microglial motility, and reduces phagocytic receptor expression triggering apoptosis/microglial phagocytosis uncoupling. PLoS Biol. 14:e1002466. doi: 10.1371/journal.pbio.1002466, PMID: 27228556 PMC4881984

[ref2] Altman J. Das G. D. (1965). Autoradiographic and histological evidence of postnatal hippocampal neurogenesis in rats. J. Comp. Neurol. 124, 319–335. doi: 10.1002/cne.901240303, PMID: 5861717

[ref3] Belenguer G. Duart-Abadia P. Jordán-Pla A. Domingo-Muelas A. Blasco-Chamarro L. Ferrón S. R.. (2021). Adult neural stem cells are alerted by systemic inflammation through TNF-α receptor signaling. Cell Stem Cell 28, 285–299.e9. doi: 10.1016/j.stem.2020.10.016, PMID: 33207218

[ref4] Ben-Hur T. (2008). immunomodulation by neural stem cells. Journal of the neurological sciences, mending the brain: stem cells and repair in multiple sclerosis. Eur. Charcot Found. Symp. 265, 102–104. doi: 10.1016/j.jns.2007.05.007 17583749

[ref5] Bielefeld P. Schouten M. Meijer G. M. Breuk M. J. Geijtenbeek K. Karayel S.. (2019). Co-Administration of Anti Micro RNA-124 and-137 oligonucleotides prevents hippocampal neural stem cell loss upon non-convulsive seizures. Front. Mol. Neurosci. 12:31. doi: 10.3389/fnmol.2019.00031, PMID: 30837840 PMC6389789

[ref6] Bonaguidi M. A. Wheeler M. A. Shapiro J. S. Stadel R. P. Sun G. J. Ming G.-l.. (2011). *In vivo* clonal analysis reveals self-renewing and multipotent adult neural stem cell characteristics. Cells 145, 1142–1155. doi: 10.1016/j.cell.2011.05.024, PMID: 21664664 PMC3124562

[ref7] Bouilleret V. Ridoux V. Depaulis A. Marescaux C. Nehlig A. La Salle G. L. G. (1999). Recurrent seizures and hippocampal sclerosis following Intrahippocampal Kainate injection in adult mice: electroencephalography, histopathology and synaptic reorganization similar to mesial temporal lobe epilepsy. Neuroscience 89, 717–729. doi: 10.1016/S0306-4522(98)00401-1, PMID: 10199607

[ref8] Bustin S. A. Benes V. Garson J. A. Hellemans J. Huggett J. Kubista M.. (2009). The MIQE guidelines: minimum information for publication of quantitative real-time PCR experiments. Clin. Chem. 55, 611–622. doi: 10.1373/clinchem.2008.112797 19246619

[ref9] Das S. Basu A. (2008). Japanese encephalitis virus infects neural progenitor cells and decreases their proliferation. J. Neurochem. 106, 1624–1636. doi: 10.1111/j.1471-4159.2008.05511.x, PMID: 18540995

[ref10] Encinas J. M. Enikolopov G. (2008). Identifying and quantitating neural stem and progenitor cells in the adult brain. Methods Cell Biol. 85, 243–272. doi: 10.1016/S0091-679X(08)85011-X 18155466

[ref11] Encinas J. M. Michurina T. V. Peunova N. Park J.-H. Tordo J. Peterson D. A.. (2011). Division-coupled astrocytic differentiation and age-related depletion of neural stem cells in the adult Hippocampus. Cell Stem Cell 8, 566–579. doi: 10.1016/j.stem.2011.03.010, PMID: 21549330 PMC3286186

[ref12] Eriksson P. S. Perfilieva E. Björk-Eriksson T. Alborn A.-M. Nordborg C. Peterson D. A.. (1998). Neurogenesis in the adult human Hippocampus. Nat. Med. 4, 1313–1317. doi: 10.1038/3305 9809557

[ref13] Ge S. Goh E. L. K. Sailor K. A. Kitabatake Y. Ming G.-l. Song H. (2006). GABA regulates synaptic integration of newly generated neurons in the adult brain. Nature 439, 589–593. doi: 10.1038/nature04404, PMID: 16341203 PMC1420640

[ref14] Hüttmann K. Sadgrove M. Wallraff A. Hinterkeuser S. Kirchhoff F. Steinhäuser C.. (2003). Seizures preferentially stimulate proliferation of radial glia-like astrocytes in the adult dentate gyrus: functional and Immunocytochemical analysis. Eur. J. Neurosci. 18, 2769–2778. doi: 10.1111/j.1460-9568.2003.03002.x, PMID: 14656326

[ref15] Kaneko N. Kudo K. Mabuchi T. Takemoto K. Fujimaki K. Wati H.. (2006). Suppression of cell proliferation by interferon-alpha through Interleukin-1 production in adult rat dentate gyrus. Neuropsychopharmacology 31, 2619–2626. doi: 10.1038/sj.npp.1301137, PMID: 16823390

[ref16] Martín-Suárez S. Abiega O. Ricobaraza A. Hernandez-Alcoceba R. Encinas J. M. (2020). Alterations of the hippocampal neurogenic niche in a mouse model of Dravet syndrome. Front. Cell Dev. Biol. 8:654. doi: 10.3389/fcell.2020.00654, PMID: 32793597 PMC7385077

[ref17] Martín-Suárez S. Valero J. Muro-García T. Encinas J. M. (2019). Phenotypical and functional heterogeneity of neural stem cells in the aged Hippocampus. Aging Cell 18:e12958. doi: 10.1111/acel.12958, PMID: 30989815 PMC6612636

[ref18] Mignone J. L. Kukekov V. Chiang A.-S. Steindler D. Enikolopov G. (2004). Neural stem and progenitor cells in nestin-GFP transgenic mice. J. Comp. Neurol. 469, 311–324. doi: 10.1002/cne.10964 14730584

[ref19] Moreno-Jiménez E. P. Flor-García M. Terreros-Roncal J. Rábano A. Cafini F. Pallas-Bazarra N.. (2019). Adult hippocampal neurogenesis is abundant in neurologically healthy subjects and drops sharply in patients with Alzheimer’s disease. Nat. Med. 25, 554–560. doi: 10.1038/s41591-019-0375-9, PMID: 30911133

[ref20] Muro-García T. Martín-Suárez S. Espinosa N. Valcárcel-Martín R. Marinas A. Zaldumbide L.. (2019). Reactive disruption of the hippocampal neurogenic niche after induction of seizures by injection of Kainic acid in the amygdala. Front. Cell Dev. Biol. 7:158. doi: 10.3389/fcell.2019.00158, PMID: 31482091 PMC6710991

[ref21] Nakanishi M. Niidome T. Matsuda S. Akaike A. Kihara T. Sugimoto H. (2007). Microglia-derived Interleukin-6 and Leukaemia inhibitory factor promote astrocytic differentiation of neural stem/progenitor cells. Eur. J. Neurosci. 25, 649–658. doi: 10.1111/j.1460-9568.2007.05309.x, PMID: 17328769

[ref22] Nayak D. Roth T. L. McGavern D. B. (2014). Microglia development and function. Annu. Rev. Immunol. 32, 367–402. doi: 10.1146/annurev-immunol-032713-120240, PMID: 24471431 PMC5001846

[ref23] Palmer T. D. Willhoite A. R. Gage F. H. (2000). Vascular niche for adult hippocampal neurogenesis. J. Comp. Neurol. 425, 479–494. doi: 10.1002/1096-9861(20001002)425:4<479::AID-CNE2>3.0.CO;2-3 10975875

[ref24] Pilz G.-A. Bottes S. Betizeau M. Jörg D. J. Carta S. April S.. (2018). Live imaging of neurogenesis in the adult mouse Hippocampus. Science, New York, N.Y. 359, 658–662. doi: 10.1126/science.aao5056, PMID: 29439238 PMC6986926

[ref25] Rodríguez-Bodero A. Encinas-Pérez J. M. (2022). Does the plasticity of neural stem cells and neurogenesis make them biosensors of disease and damage? Front. Neurosci. 16:977209. doi: 10.3389/fnins.2022.977209, PMID: 36161150 PMC9493188

[ref26] Rolls A. Shechter R. London A. Ziv Y. Ronen A. Levy R.. (2007). Toll-like receptors modulate adult hippocampal neurogenesis. Nat. Cell Biol. 9, 1081–1088. doi: 10.1038/ncb1629, PMID: 17704767

[ref27] Schindelin J. Arganda-Carreras I. Frise E. Kaynig V. Longair M. Pietzsch T.. (2012). Fiji: an open-source platform for biological-image analysis. Nat. Methods 9, 676–682. doi: 10.1038/nmeth.2019, PMID: 22743772 PMC3855844

[ref28] Sierra A. Encinas J. M. Deudero J. J. P. Chancey J. H. Enikolopov G. Overstreet-Wadiche L. S.. (2010). Microglia shape adult hippocampal neurogenesis through apoptosis-coupled phagocytosis. Cell Stem Cell 7, 483–495. doi: 10.1016/j.stem.2010.08.014, PMID: 20887954 PMC4008496

[ref29] Sierra A. Martín-Suárez S. Valcárcel-Martín R. Pascual-Brazo J. Aelvoet S.-A. Abiega O.. (2015). Neuronal hyperactivity accelerates depletion of neural stem cells and impairs hippocampal neurogenesis. Cell Stem Cell 16, 488–503. doi: 10.1016/j.stem.2015.04.003, PMID: 25957904 PMC4443499

[ref30] Song J. Zhong C. Bonaguidi M. A. Sun G. J. Hsu D. Yan G.. (2012). Neuronal circuitry mechanism regulating adult quiescent neural stem cell fate decision. Nature 489, 150–154. doi: 10.1038/nature11306, PMID: 22842902 PMC3438284

[ref31] Sullivan P. G. Keller J. N. Mattson M. P. Scheff S. W. (1998). Traumatic brain injury alters synaptic homeostasis: implications for impaired mitochondrial and transport function. J. Neurotrauma 15, 789–798. doi: 10.1089/neu.1998.15.789, PMID: 9814635

[ref32] Sultan S. Li L. Moss J. Petrelli F. Cassé F. Gebara E.. (2015). Synaptic integration of adult-born hippocampal neurons is locally controlled by astrocytes. Neuron 88, 957–972. doi: 10.1016/j.neuron.2015.10.037, PMID: 26606999

[ref33] Sun L. (2004). Neuronally expressed stem cell factor induces neural stem cell migration to areas of brain injury. J. Clin. Investig. 113, 1364–1374. doi: 10.1172/JCI200420001, PMID: 15124028 PMC398428

[ref34] Sun G. Q. Yu R. T. Evans R. M. Shi Y. (2007). Orphan nuclear receptor TLX recruits histone deacetylases to repress transcription and regulate neural stem cell proliferation. Proc. Natl. Acad. Sci. 104, 15282–15287. doi: 10.1073/pnas.0704089104, PMID: 17873065 PMC2000559

[ref35] Valcárcel-Martín R. Martín-Suárez S. Muro-García T. Pastor-Alonso O. Rodríguez F. de Fonseca G.. (2020). Lysophosphatidic acid receptor 1 specifically labels seizure-induced hippocampal reactive neural stem cells and regulates their division. Front. Neurosci. 14:811. doi: 10.3389/fnins.2020.00811, PMID: 32922255 PMC7456947

[ref36] Widera D. Mikenberg I. Elvers M. Kaltschmidt C. Kaltschmidt B. (2006). Tumor necrosis factor α triggers proliferation of adult neural stem cells via IKK/NF-κB signaling. BMC Neurosci. 7:64. doi: 10.1186/1471-2202-7-64, PMID: 16987412 PMC1586209

[ref37] Zheng L.-S. Hitoshi S. Kaneko N. Takao K. Miyakawa T. Tanaka Y.. (2014). Mechanisms for interferon-α-induced depression and neural stem cell dysfunction. Stem Cell Rep. 3, 73–84. doi: 10.1016/j.stemcr.2014.05.015, PMID: 25068123 PMC4110771

