# Supplementary material for: The differential response to neuronal hyperexcitation and neuroinflammation of the hippocampal neurogenic niche
Source: Front Neurosci. 2023 Jul 11;17:1186256. doi: 10.3389/fnins.2023.1186256 (PMC10366379; doi:10.3389/fnins.2023.1186256)
Supplement: Supplementary file 1 [file Data_Sheet_1.pdf]

Supplementary Figures

Supplementary Figure 1. Statistical results for 3D-Sholl analysis. Level of significance corresponding to the comparison among all groups for each point of analysis (radius)* $p<0.05$, **$p < 0.01$, ***$p <0.001$.

Supplementary Figure 2. TBI induces C-Fos expression. (A) Confocal microscopy images showing immunostaining for C-Fos and counterstaining for DAPI in the dentate gyrus. **(B)** Quantification of the density of C-Fos positive cells in the GCL in NI models, TBI and corresponding controls 3 days after the application of the stimulus. Scale bar is 50 µm. * $p<0.05$, **$p < 0.01$, ***$p <0.001$ one-way ANOVA after all pairwise multiple comparisons by Holm-Sidak post hoc test. Bars show mean ± SEM. Dots show individual data.

Supplementary Figure 3. NI models trigger microgliosis. (A) Confocal microscopy images of the dentate gyrus after immunostaining for the microglia marker Iba-1 and BrdU. DAPI was used for counterstaining. Changes in microglia morphology can be observed in all models. **(B)** Quantification of the proportion of microglia immunospositive for BrdU. See Supplementary Figure 4A for statistical results. **(C)** Quantification of the proportion of change of CD68 mRNA. See Supplementary Figure 4B for statistical results. **(D)** Quantification of the proportion of change of IL-1β mRNA. See Supplementary Figure 4C for statistical results. **(E)** Quantification of the proportion of change of TNF-α mRNA **(F)** Proportion of dividing NSCs among the total population of NSCs (NI vs control). See Supplementary Figure 4A for statistical results. See Supplementary Figure 4D for statistical results.

Supplementary Figure 4. Statistical results for microglial proliferation (A) and mRNA for CD68 (B), IL-1β (C) and TNF-α (D). Level of significance corresponding to the comparison among all groups for each point of analysis (radius)* $p<0.05$, **$p < 0.01$, ***$p <0.001$, after one-way ANOVA after all pairwise multiple comparisons by Holm-Sidak post hoc test.
