# Supplementary figures and images for: The differential response to neuronal hyperexcitation and neuroinflammation of the hippocampal neurogenic niche
Source: Front Neurosci. 2023 Jul 11;17:1186256. doi: 10.3389/fnins.2023.1186256 (PMC10366379; doi:10.3389/fnins.2023.1186256)

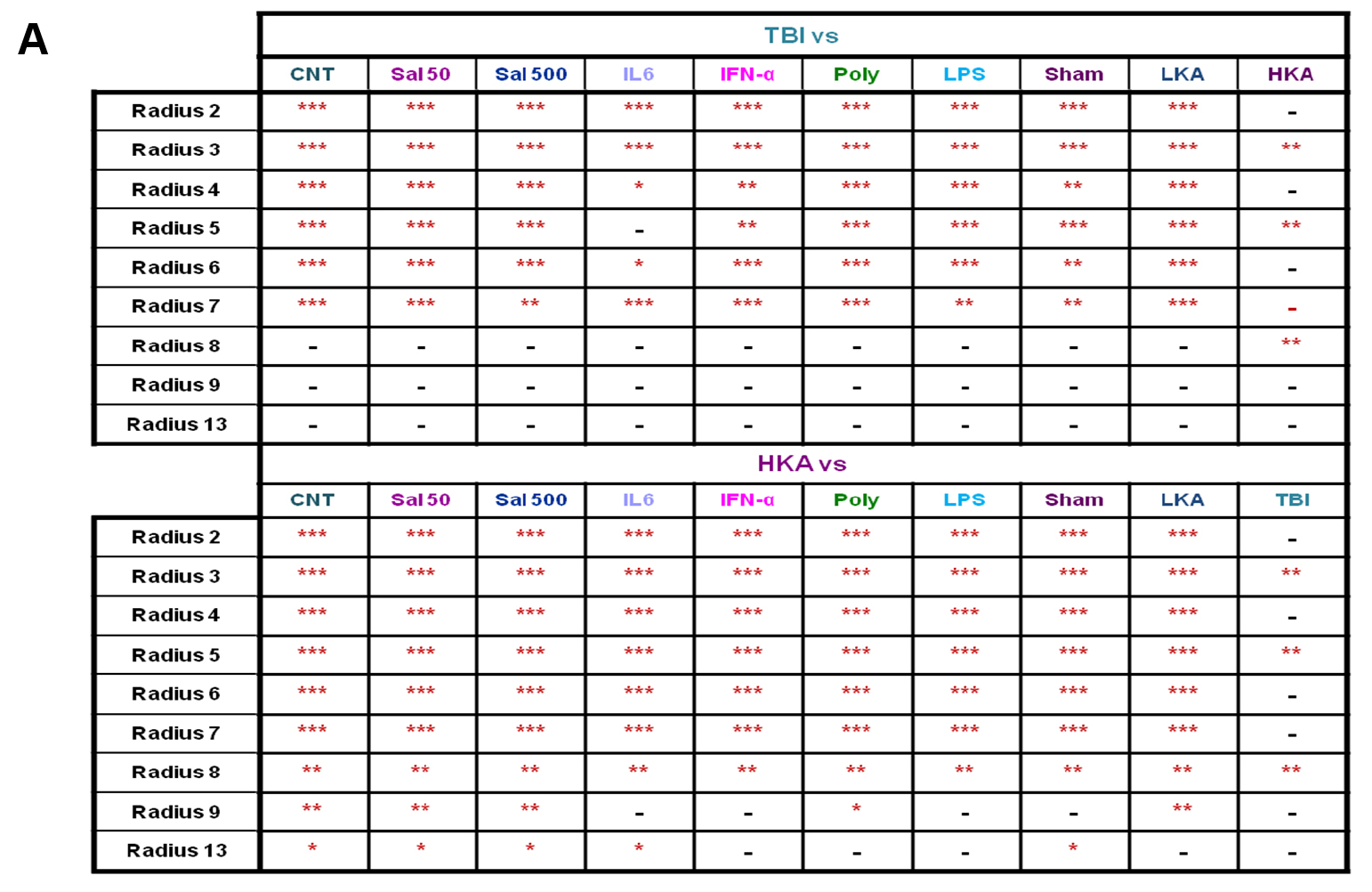

Supplement: Supplementary file 2 [file Image_1.jpg]

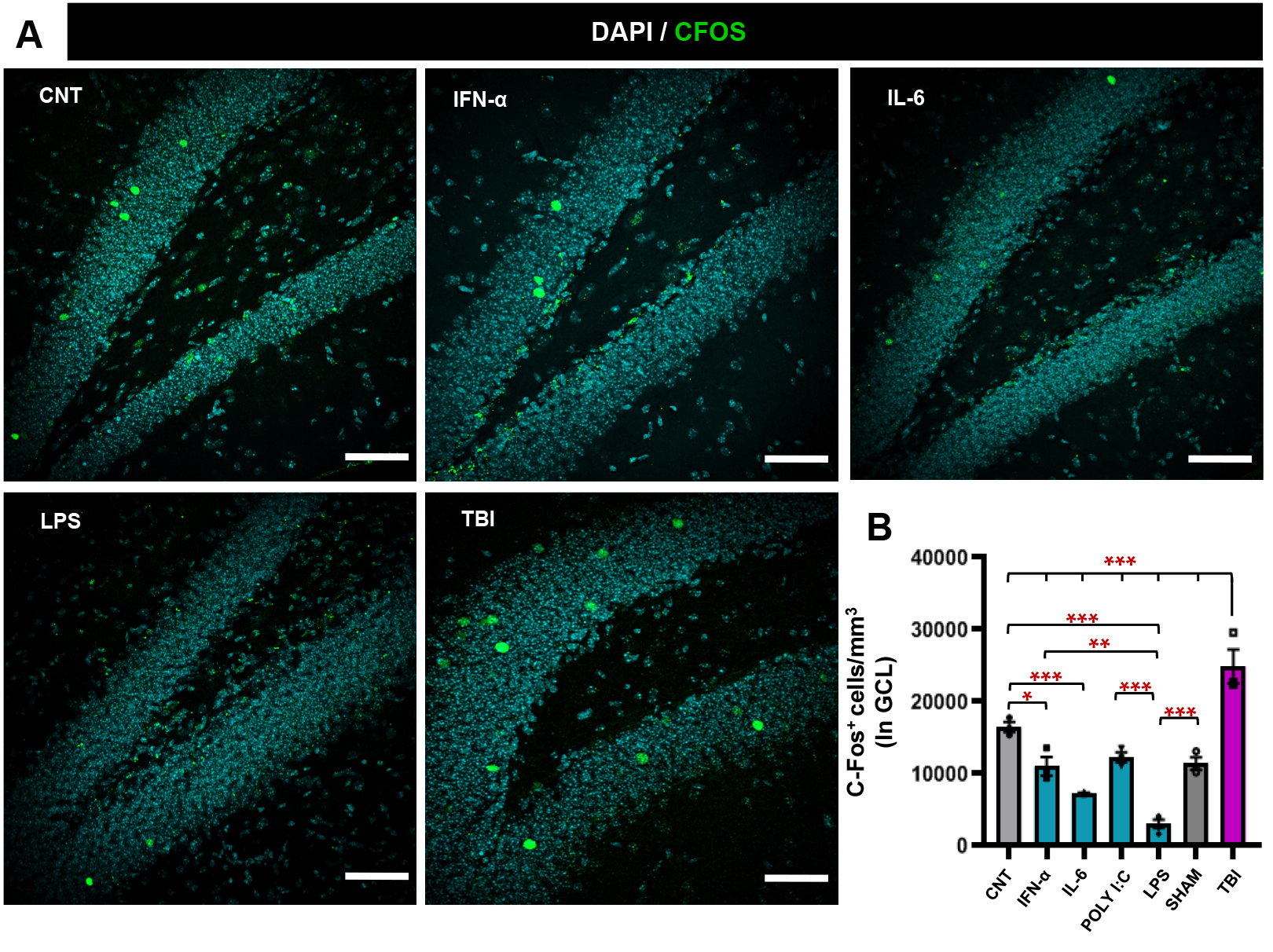

Supplement: Supplementary file 3 [file Image_2.jpg]

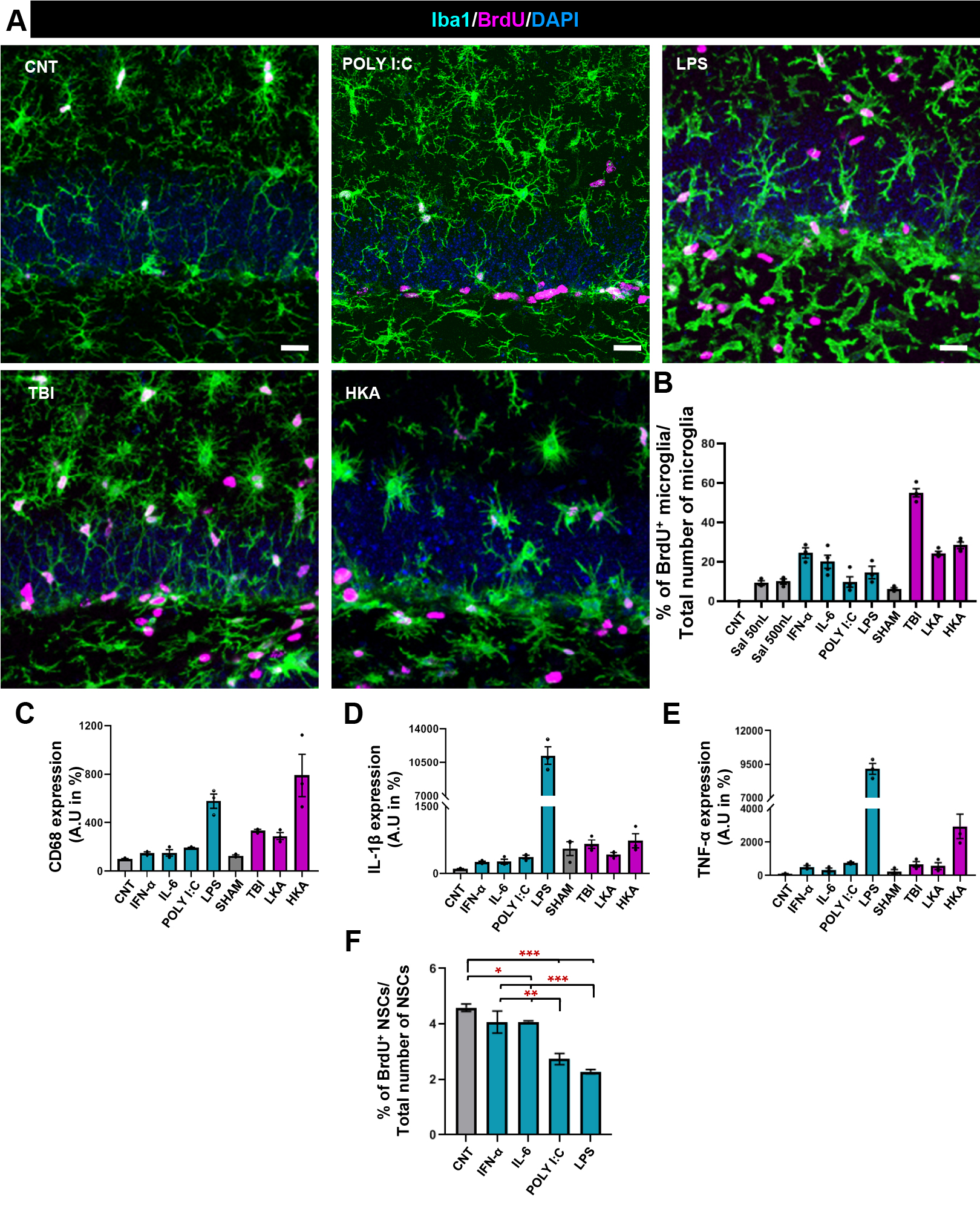

Supplement: Supplementary file 4 [file Image_3.jpg]

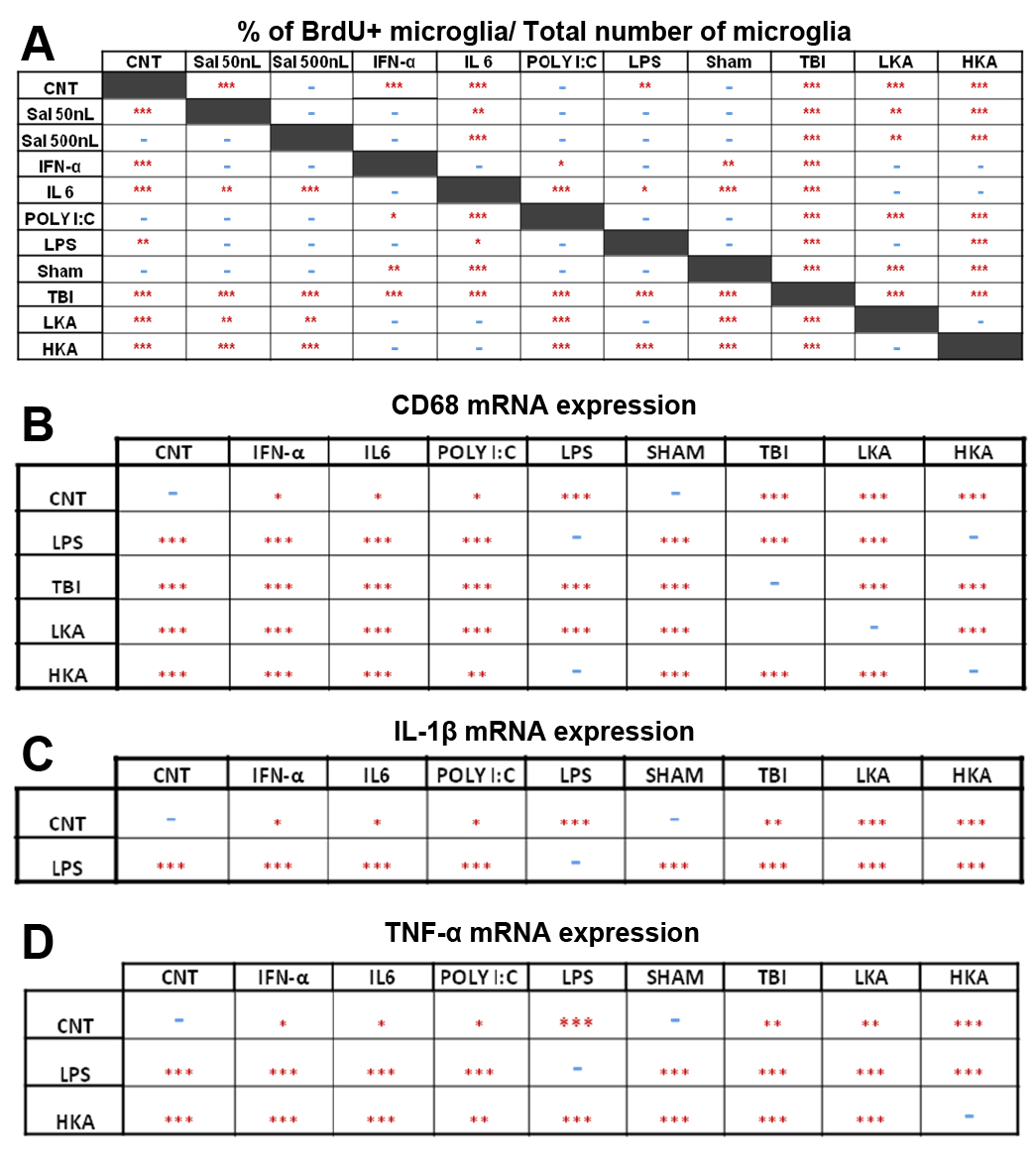

Supplement: Supplementary file 5 [file Image_4.jpg]
